# Differential Network Analysis Applied to Preoperative Breast Cancer Chemotherapy Response

**DOI:** 10.1371/journal.pone.0081784

**Published:** 2013-12-09

**Authors:** Gregor Warsow, Stephan Struckmann, Claus Kerkhoff, Toralf Reimer, Nadja Engel, Georg Fuellen

**Affiliations:** 1 Institute for Biostatistics and Informatics in Medicine and Ageing Research, University of Rostock Medical School, Rostock, Germany; 2 Department of Mathematics and Informatics, University of Greifswald, Greifswald, Germany; 3 Department of Anatomy and Cell Biology, University Medicine Greifswald, Greifswald, Germany; 4 Department of Biomedical Sciences, School of Human Sciences, University of Osnabrueck, Germany; 5 Department of Obstetrics and Gynecology, University of Rostock Medical School, Germany; 6 Department of Cell Biology, University of Rostock Medical School, Rostock, Germany; Leibniz-Institute for Farm Animal Biology (FBN), Germany

## Abstract

In silico approaches are increasingly considered to improve breast cancer treatment. One of these treatments, neoadjuvant TFAC chemotherapy, is used in cases where application of preoperative systemic therapy is indicated. Estimating response to treatment allows or improves clinical decision-making and this, in turn, may be based on a good understanding of the underlying molecular mechanisms. Ever increasing amounts of high throughput data become available for integration into functional networks. In this study, we applied our software tool ExprEssence to identify specific mechanisms relevant for TFAC therapy response, from a gene/protein interaction network. We contrasted the resulting active subnetwork to the subnetworks of two other such methods, OptDis and KeyPathwayMiner. We could show that the ExprEssence subnetwork is more related to the mechanistic functional principles of TFAC therapy than the subnetworks of the other two methods despite the simplicity of ExprEssence. We were able to validate our method by recovering known mechanisms and as an application example of our method, we identified a mechanism that may further explain the synergism between paclitaxel and doxorubicin in TFAC treatment: Paclitaxel may attenuate MELK gene expression, resulting in lower levels of its target MYBL2, already associated with doxorubicin synergism in hepatocellular carcinoma cell lines. We tested our hypothesis in three breast cancer cell lines, confirming it in part. In particular, the predicted effect on MYBL2 could be validated, and a synergistic effect of paclitaxel and doxorubicin could be demonstrated in the breast cancer cell lines SKBR3 and MCF-7.

## Introduction

### Breast cancer and network-based approaches

For the successful treatment of breast cancer, the most common type of cancer in women worldwide, knowledge of cancer-treatment responsiveness is most useful. Substantial progress was made in understanding disease mechanisms of breast cancer, but many questions are still unanswered. The rise of genome-scale gene expression profiling allowed for identification of biomarkers that help to further subcategorize known groups of breast cancer, among them luminal (ER^+^/HER2^−^), HER2-enriched (HER2^+^) and triple-negative (ER^−^/PR^−^/HER2^−^) types.

Profiling approaches were first based on the identification of single, differentially expressed genes or of gene sets (signatures). Nowadays, research follows an integrative approach utilizing gene/protein interaction networks, thereby reflecting that biological processes are performed by genes/proteins/molecules interacting with each other and not acting individually [1]–[9]. Some specific approaches are detailed below. Especially for breast cancer, the utilization of subnetworks instead of single genes as biomarkers has been suggested as they provide higher prediction accuracy for both prognosis and classification purposes [10], [11], even though the value of network-based methods is still a matter of debate [12]. In terms of complexity, network-based approaches go beyond former analysis methods, as the number of genes in the human genome is surprisingly low (around 23,000 protein coding genes), but the number of interactions and dependencies between them allows for a large variety of processes in the cell.

### Working Hypothesis of our Approach

The work presented here attempts to extract the molecular mechanisms that are relevant for successful chemotherapeutical breast cancer treatment from a gene/protein interaction network. More precisely, our work hypothesis is that our method ExprEssence can use gene expression data to extract a subnetwork from an all-purpose gene/protein interaction network, which includes some of the most important mechanisms related to the differences between responders and non-responders to TFAC therapy.

### Input data and related approaches

Specifically, we used an all-purpose gene/protein interaction network based on the STRING database [13], into which large genome-scale datasets, assembled from more than 200 patients from various breast cancer subtypes [14] were integrated. Patient collectives of this size enable unprecedented statistical power and robustness despite subgroup differences. We applied our previously published method ExprEssence [15] to identify altered gene/protein interactions that characterize the differences between the responders and non-responders to neoadjuvant TFAC therapy. We assume these differentially regulated interactions to be related or even critical for therapy outcome. Knowing about the differences between responders and non-responders may help to gain more detailed insights into both the progression of breast cancer and how it is affected by drugs, which is of high relevance for choosing individualized cancer treatment.

Besides ours, there are several network-based approaches aiming to identify genes or proteins involved in the response to a treatment or external condition [3], [4], [7], including the pioneering work of Ideker et al. [1]. We compare the results of our method to two such methods, OptDis [7] and KeyPathwayMiner [9], [16] investigating the same breast cancer dataset by all methods. We find that ExprEssence generates subnetworks more directly associated with disease- and drug-related processes than the other methods. Furthermore, using the subnetwork extracted by ExprEssence, we inferred a hypothesis about a mechanism putatively contributing to TFAC mode of action in chemotherapy, and we experimentally validated it in part.

## Materials and Methods

### In silico a nalyses

#### Gene/protein interaction network and gene expression data

The interaction network, from which the subnetworks of ExprEssence and KeyPathwayMiner were extracted, was based on the STRING database, version 9.0 [13]. It contained all human interactions scoring at least 0.85 for experimental, database or textmining evidence channels.

We used breast cancer therapy -related gene expression data from the MicroArray Quality Control (MAQC)-II study ([14], GSE20194). The data were generated at the MD Anderson Cancer Center (MDACC, Houston TX, USA). In this study, transcriptome data as well as ER, PR and HER2 receptor status of 230 patients with newly diagnosed breast cancer were acquired by fine-needle aspiration before neoadjuvant chemotherapy with TFAC (a combination of paclitaxel (**T**axol®), 5-**f**luorouracil, doxorubicin (**A**driamycin®) and **c**yclophosphamide). The patients were classified as responders (48) or non-responders (182) after tumor resection. The gene expression data of all 230 specimen were collected into a table and were quantile normalized. For each probe set identifier represented on the array, we averaged the individual expression measures group-wise to obtain single probe set level expression values for the responders and non-responders, respectively. Using the Affymetrix annotation file, the probe set identifiers were then mapped to UniGene identifiers. When several probe set identifiers matched to the same UniGene identifier, its gene expression value was calculated as the mean expression of the respective probe sets. The gene expression data were integrated into the interaction network using UniGene identifiers for mapping.

#### Subnetwork detection methods

**Figure 1:**
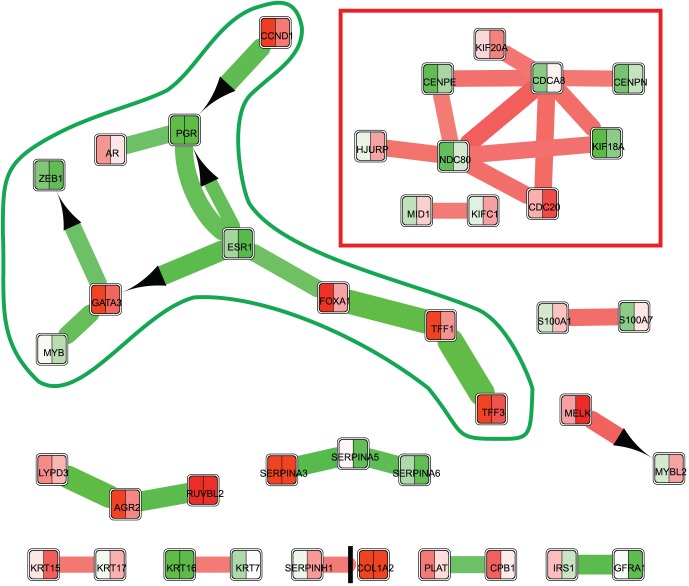
ExprEssence-condensed network describing the 16 most and 16 least active interactions between the E40 genes/proteins. For each gene, its mean expression level is visualized for non-responders (left) and responders (right) by color (green for low, white for intermediate, red for high expression). Interactions between the genes/proteins are represented by a line. Stimulations are indicated by an arrow on the target, inhibitions by a t-bar. The up- (red) and down-regulation (green) of interactions are also colorcoded. Full gene names can be found in Table S1.

In this work, we utilized three subnetwork detection tools, as follows. The O39 gene set generated by the OptDis tool [7] was compared to the results of two other subnetwork-detecting tools: our tool ExprEssence [15] and the tool KeyPathwayMiner (v4.0) [9], [16], both available as plugins for the Cytoscape platform [17]. In contrast to most other methods for active subnetwork detection, including OptDis and KeyPathwayMiner, ExprEssence does not focus on retrieving connected components but on identifying single interactions that are regulated most differentially among all interactions in the original network. Nevertheless, the genes in an ExprEssence subnetwork often aggregate into several connected components (see the red and green frames in Figure 1), reflecting the biological relevance of the identified components in the subnetwork.

- **OptDis.** The gene expression data just described was used by Dao et al. for the application of their OptDis method, which generates subnetworks that are most suitable for the distinction between two conditions (responders and non-responders) [7]. In their study, the 230 cases were split up into a discovery and a validation group. After applying OptDis on both sets individually, they intersected the gene sets that made up the respective top-50 subnetworks for each group (discovery and validation). The overlap, a set of 39 genes (denoted as O39), was used for an Ingenuity® IPA Functional Enrichment Analysis.
- **ExprEssence.** ExprEssence generates a subnetwork by choosing those interactions of a gene/protein interaction network that are most differentially regulated between responders and non-responders. We integrated the gene expression data (described above) into the interaction network (also described above; the gene expression data mapped to 9410 genes of the network), and used it as input for ExprEssence. ExprEssence then determined the link score for each interaction, which describes the amount and direction of differential regulation of the interaction. Afterwards, ExprEssence was used to construct a subnetwork of the interaction network, comprising those interactions with highest amount of change between responders and non-responders, that is of those interactions with largest absolute values of the link scores. For details on the link score calculation, see Warsow et al. [15]. The number of most differentially regulated interactions in the subnetwork can be altered by the user. To obtain a subnetwork with a number of genes comparable to the O39 gene set, we retrieved the 16 most up- and 16 most down-regulated interactions, resulting in a subnetwork with 40 genes (denoted as E40), see Figure 1.
- **KeyPathwayMiner.** For subnetwork generation with KeyPathwayMiner, an indicator matrix had to be supplied that defines which genes are "active", e.g. differentially regulated, in which of the responder samples. In order to generate this indicator matrix, we followed procedures as recommended by the authors, as follows. The mean expression and its variance were calculated gene-wise for all non-responders after normalization (to a gene expression of mean 0 and variance 1 for each sample). The single expression values of each responder were then tested against the respective mean and variance from the non-responders to obtain the significantly differentially expressed genes (on a significance level of 5%). The resulting matrix of zeros and ones described which genes were differentially expressed in which responder (value 1), and which ones were not (value 0).

In contrast to ExprEssence, which does not enforce the interactions of the subnetwork to be connected, KeyPathwayMiner aims for finding maximal connected subgraphs (maximal with respect to the number of genes). Furthermore, each gene of the subgraph has to be active (e.g. differentially expressed) in all but at most *L* cases, and each subgraph must contain at most *K* non-active genes. In this study, the cases were the responders, whose gene expression is put into relation with the gene expression of all non-responders. Using the network and the indicator matrix, the KeyPathwayMiner subnetworks were then calculated using the Ant Colony Optimization (ACO) search algorithm and the individual node exceptions (INES; genes are represented by nodes in the network) search strategy. The default ACO advanced parameters were not changed. The node exceptions parameter *K* was set to the default value of 8, and the case exceptions parameter L was set to 38 based on initial analysis results (see Results and Discussion).

#### IPA Analyses

The genes comprising the ExprEssence and KeyPathwayMiner subnetworks, respectively, were then used for an IPA Functional Enrichment Analysis (using the Ingenuity database as of 09/01/2013) to compare the results with the IPA analysis of the O39 gene set. Due to updates in the database underlying IPA, the IPA analysis for the O39 gene set was redone to allow for a fair direct comparison of the IPA results of all three methods (OptDis, ExprEssence and KeyPathwayMiner).

### Cell culture and treatment conditions

Breast cancer cell lines MCF-7, BT-20 and SKBR3 and MCF-10A cells were obtained from American Type Culture Collection (ATCC, USA). MCF-7 and BT-20 were maintained in Dulbecco's modified Eagle's medium (Invitrogen, Germany) with 10% fetal bovine serum (PAN Biotech GmbH, Germany) and 1% gentamycin (Ratiopharm, Germany). SKBR3 cell line was cultured in McCoy's 5a Medium (ATCC, USA) supplemented with 10% fetal bovine serum (PAN Biotech GmbH, Germany) and 1% gentamycin (Ratiopharm, Germany). The non-tumorigenic control cell line MCF-10A was grown in Dulbecco's modified Eagle's medium Ham's F12 without phenol red (Invitrogen, Germany) containing 10% horse serum (PAA Laboratories GmbH, Germany), the Mammary Epithelial Cell Growth Medium Supplement Pack (Promo Cell, Germany) including Bovine Pituitary Extract (0.004 ml/ml), Epidermal Growth Factor (recombinant human) 10 ng/ml, Insulin (recombinant human) 5 µg/ml, Hydrocortisone 0.5 µμg/ml and 1% gentamycin (Ratiopharm, Germany). All cell lines were authenticated by morphology and growth rate and were mycoplasma free. Prior treatment, all cell lines were seeded in 6-well plates and adapted to phenol-red-free Dulbecco's modified Eagle's medium (PAA Laboratories GmbH, Germany) with 10% charcoal stripped fetal bovine serum (PAN Biotech GmbH, Germany) for 48 h (assay medium). Paclitaxel (T, Taxol; Ratiopharm, Germany) at a final concentration of 0.1 nM or 0.1 µM, doxorubicin hydrochloride (A, Adriamycin; Sigma, Germany) at a final concentration of 1 nM or 1 µM, or both were added to the cells for 24 or 48 h in fresh assay medium. As negative control the diluent EtOH (0.1%) was used in the same manner.

### Western blot

After treatment with T or/and A or rather with the control substance EtOH for at least 48 h, the cells were trypsinized, washed with PBS and lysed in ice-cold lysis buffer (Bio-Plex Cell Lysis Kit, Bio-Rad, USA). Cells were homogenized by brief sonification at 4°C and centrifuged at 8,000 *g* for 1 min at 4°C. Protein concentrations of supernatants were estimated by Bradford protein assay [18] so that equal amounts (10–20 µg) of total soluble protein could be separated by Criterion TGX Stain-Free precast gels (Bio-Rad, Germany) and blotted on PVDF membranes. After SDS-PAGE, protein content per lane as well separation quality was additionally controlled with the Criterion Stain FreeTM gel imaging system (Bio-Rad, Germany). Protein transfer was carried out with a tank blotting system (Bio-Rad, Germany) and then, membranes were blocked with 5% skim milk in Tris-buffered saline (TBS) and washed five times in TBS. For protein detection, primary antibodies (anti-rabbit anti-MYBL2, AP20207PU-N, Acris, USA; anti-rabbit anti-Melk, 2274, Cell signaling, USA; anti-mouse anti-PCNA, sc-56, Santa Cruz, USA; anti-mouse anti-Actin, sc-47778, Santa Cruz, USA) were incubated overnight at 4°C followed by a labeling with a horseradish peroxidase (HPR)-conjugated secondary antibody (Dako, Glostrup, Denmark) for 1 h at room temperature. Protein signals were visualized by using SuperSignal West Femto Chemiluminescent Substrate (Pierce Biotechnology, Rockford, USA) for detection of peroxidase activity. Band intensity was analyzed densitometrically with the Molecular Imager ChemiDoc XRS and Image Lab 3.0.1 software (Bio-Rad, USA). Protein detection was repeated at a minimum of three times with individually prepared cell lysates from independent passaged cells.

### MTS assay

Cells seeded in 96-well-plates in 100 µL medium were treated with indicated compounds. After 48 h incubation at 37°C in a 5% CO_2_ atmosphere, cells were assayed with 10 µL MTS [3-(4,5 dimethylthiazol-2-yl)-5-(3-carboxymethoxyphenyl)-2-(4-sulfophenyl)-2H-tetrazolium] solution (Promega Corp., Madison, WI) for 1 h at 37°C. The vehicle EtOH (0.1%) was used in the same manner to serve as control. Colorimetric changes were measured at 492 nm and correction for background absorbance was done by measuring the absorbance of the compounds and MTS solution without the cells. Raw data were transferred to Microsoft Excel for analysis.

### Live-Dead Assay

Live-Dead Assay was carried out following manufacturer's instructions (PromoCell GmbH, Heidelberg, Germany). After treatment with the indicated compounds cells were washed with phosphate buffered saline to remove serum esterase activity and then treated with 200 µL of Calcein AM/Ethidium homodimer-lll (EthD-lll) standard working solution. Cells were incubated at 37°C for 1 h (Promo cell GmbH, Heidelberg, Germany). The percentage of stained live and dead cells was measured by a fluorescence multiplate reader (Tecan M200, GmbH, Austria) at appropriate wavelengths; Calcein AM (Ex/Em ∼495/∼515 nm), EthD-lll (Ex/Em ∼530/∼635 nm). The relative reference to the cell number was ensured by a simultaneous Hoechst staining. All obtained values were normalized with respect to the cell number.

### Cell cycle measurement for proliferation analysis

To determine proliferation, cell cycle analysis was performed by flow cytometry [9]. The software FlowJo version 10.0.5 (Tree Star Inc., USA) was used to acquire data. A minimum of 15,000 ungated events were recorded. Double and clumps were excluded by gating on the DNA pulse width versus pulse area displays. For statistical analysis, the S-phase and G2/M-phase cells were defined as proliferative cells.

## Results and Discussion

### Generation of active subnetworks by three methods

In this study, we applied our active interaction/subnetwork detection method ExprEssence to the investigation of response status to breast cancer chemotherapy with TFAC, and we compared the results to two similar methods, OptDis and KeyPathwayMiner.

In the OptDis study by Dao et al., a set of 39 genes was deemed to be of high importance for differences between responders and non-responders to TFAC therapy (see Materials and Methods section). An IPA Functional Enrichment Analysis was performed for the O39 gene set. We performed the same analysis on the subnetworks extracted through application of ExprEssence and KeyPathwayMiner on the same network using the same gene expression data. KeyPathwayMiner was chosen for comparison with ExprEssence, as it is a recently published method that has been shown to outperform other active subnetwork detection methods (GiGA [2], CUSP [5] and jActiveModules [1]). Like an ExprEssence subnetwork, a KeyPathwayMiner network is easily interpretable.

The ExprEssence subnetwork was generated such that it contains approximately the same number of genes as the O39 gene set. Hence, this network contains the 16 most up- and 16 most downregulated interactions, encompassing 40 genes (E40 ; the gene names are listed in Table S1). Further parameters were not required for the ExprEssence analysis.

For KeyPathwayMiner, we used the parameter settings as described in Materials and Methods. We first set the case exceptions parameter *L* to 10 (i.e., the recommended one fifth of the number of cases (48)) but we obtained an empty network. This is most likely due to the inhomogeneity of gene expression among the responders; no single gene is differentially expressed (active) with respect to the non-responders in 15 or more responders. We found that the parameter *L* had to be set to at least 34 in order to allow 5 genes to be included into a subnetwork. Using higher values for *L* leads to the incorporation of more genes (which are then active in fewer cases), and we settled for a value of *L* = 38 to obtain 20 subnetworks, each containing 28 genes. Using an even higher case exception parameter setting gave us limited returns in terms of increases in subnetwork size, while moving us away from an adequate use of KeyPathwayMiner. For each of the top 5 KeyPathwayMiner subnetworks (Figures S1, S2, S3, S4 and S5), an IPA analysis was performed. The 5 subnetworks strongly overlap (see Table S2), as do their IPA analysis results (see Table S3). Therefore, in Table 1, the top 25 functional terms resulting from the IPA analysis are shown only for the genes of the first KeyPathwayMiner subnetwork (KPM1) together with the results for the E40 and O39 gene sets.

**Table 1:** The top 25 terms of Ingenuity Functional Enrichment Analysis for the genes found by ExprEssence (E40), the genes found by OptDis (O39) and the genes found by KeyPathwayMiner (KPM1 network).

	Enriched Functional Terms	
E40 Genes	O39 Genes	KPM1 Network Genes
Cell cycle progression	Transactivation of RNA	Differentiation of cells
Breast cancer	Development of tumor	Proliferation of neuronal cells
Carcinoma in breast	Cell cycle progression	Migration of neural crest cells
Chromosomal congression of chromosomes	Cell movement	Cell movement
Amenorrhea	Proliferation of tumor cell lines	Development of central nervous system
Digestive organ tumor	Necrosis	Migration of cells
Proliferation of cells	Transcription	Transactivation of RNA
Metrorrhagia	Proliferation of connective tissue cells	Expression of RNA
Plaque psoriasis	Migration of cells	Transcription of RNA
Proliferation of tumor cells	Apoptosis of tumor cell lines	Proliferation of cells
Cell movement	Transcription of RNA	Development of autonomic nervous system
Uterine hemorrhaging	Proliferation of cells	Transcription of DNA
Proliferation of breast cancer cell lines	Cell survival	Apoptosis
M phase	Cell death	Abnormal morphology of embryonic tissue
Invasion of tumor cell lines	Cell death of tumor cell lines	Development of brain
Triple-negative breast cancer	Proliferation of epithelial cells	Differentiation of muscle cells
Cancer	Differentiation of cells	Development of lymphatic system component
Cell cycle progression of tumor cell lines	Hypoplasia	Morphology of head
Mitosis	Cell viability	Morphology of nervous system
Organization of cytoskeleton	Apoptosis	Activation of DNA endogenous promoter
Invasion of cells	Synthesis of DNA	Development of cerebellum
Skin development	Proliferation of fibroblasts	Development of body axis
Development of epidermis	Binding of DNA	Abnormal morphology of endolymphatic duct
Epithelial neoplasia	Quantity of cells	Proliferation of epithelial cells
Gastrointestinal Tract Cancer and Tumors	Abnormal morphology of embryonic tissue	Cell death

Table S4 and Table S5 contain also p -values and the lists of the genes associated with the terms.

### Comparison of the subnetworks based on functional enrichment

According to the IPA Functional Enrichment Analysis results (Table 1) of the gene sets derived from the three subnetworks, the gene set being most associated with breast cancer chemotherapy is the ExprEssence E40 gene set, followed by the OptDis O39 gene set and the one derived from the top KeyPathwayMiner network (KPM1). In fact, the E40 gene set does not just feature more TFAC therapy related terms such as *Chromosomal congression of chromosomes*, *M phase* or *Mitosis* among the top 25 enriched terms, but also more breast cancer terms, among them *breast cancer* itself, *Carcinoma in breast*, *Proliferation of breast cancer cell lines* and *Triple-negative breast cancer* (Table 1). Therefore, compared to the gene set based on KeyPathwayMiner and the O39 gene set, the E40 gene set is more specific with respect to the biological mechanisms that distinguish responders from non-responders with respect to breast cancer treatment with TFAC. ExprEssence directly determines a score for each interaction, describing the direction and amount of change in interaction strength between the groups of responders and non-responders. KeyPathwayMiner, however, uses the interaction network to connect as many active genes as possible to obtain a maximal connected subgraph. Consequently, using ExprEssence, a link may be deemed important, even if the genes connected by the link are not significantly differentially expressed. In fact, only 85% of the genes in the E40 gene set are significantly differentially expressed. However, all interactions except one (KRT16-KRT7) are differentially regulated in a statistically significant way with Benjamini-Hochberg adjusted P-values below 0.05 for the two-tailed t-test. ExprEssence thus picks up relevant genes due to interactions between their respective proteins, without taking into account whether the differential regulation of the individual genes is significant. In the next section, we will discuss the subnetwork identified by ExprEssence in more detail to investigate our work hypothesis, i.e. to further demonstrate that this subnetwork includes some of the most important mechanisms related to the differences between responders and non-responders to TFAC therapy.

### The ExprEssence-condensed network

In this section, the most relevant findings by ExprEssence (Figure 1) will be discussed, consisting of interactions that are regulated most differentially between responders and non-responders to TFAC chemotherapy. Liedtke et al. [20] reported that triple-negative breast cancer is responding better to neoadjuvant chemotherapy compared to other types, especially compared to ER-positive breast cancer. In turn, one may expect a large proportion of TFAC responders to feature low expression of the ER receptor. This indeed is reflected in our subnetwork (green frame), showing lower ESR1 (as well as AR and PGR) expression in the group of responders compared to non-responders.

More generally, in our analysis, interactions involving these receptors describe some of the major differences between responders and non-responders. They build up parts of the cluster boxed in green, which contains interactions that are downregulated most strongly in responders compared to non-responders. In particular, this cluster includes the mutual stimulation between GATA3 and ESR1. This interaction has been hypothesized by Eeckhoute et al. [21] to promote breast cancer progression via a positive cross-regulatory loop. Despite its cancer promoting role, expression of GATA3 is indicative for good general prognosis [22], as it is strongly correlated with ESR1 expression [23] and such cancer cells can be treated with high rates of success with hormone therapy, but not TFAC therapy [20]. In summary, we successfully identified the downregulated interaction between GATA3 and ESR1 to be beneficial for good TFAC response, even though prognosis is worse in general for triple-negative breast cancer (low ESR1 expression) compared to hormone therapy-treatable receptor positive breast cancers (higher ESR1 expression).

The interactions within the red frame in Figure 1 are upregulated in responders compared to non-responders. These interactions occur exclusively between genes/proteins associated with cell cycle and mitosis. Cells of responders are thus mitotically more active than non-responder cells, allowing the mitotic spindle poison paclitaxel to have a stronger therapeutical effect [24]. A detailed discussion of other interactions in Figure 1 can be found in the Text S1, and in the next section, we will describe the general pattern we find in the role of upregulated and downregulated interactions.

### General patterns in the ExprEssence subnetwork

Motivated by the parts of the subnetwork that are highlighted by the green and red frames, we can interpret up- and down-regulated interactions in the ExprEssence subnetwork in the context of TFAC therapy, as follows. We observe that links that are *upregulated* in responders (interactions represented by red lines in Figure 1) can be divided into two classes: The first class supports *tumorsuppressive* mechanisms (indicating good prognosis irrespective of treatment). The other class supports *pro-oncogenic* mechanisms (e.g. cell cycle/mitosis related links). Here, each such interaction itself or at least one of the proteins involved in it is known as a target of TFAC. In turn, non-responders do not feature these specific pro-oncogenic targets for chemotherapy and therefore cannot benefit from the therapy as much. Therefore, we suppose that upregulated pro-oncogenic processes are targets for therapy and hence a basis for TFAC response.

*Downregulated* links, on the other hand, cannot be associated closely to response to TFAC. Instead, if the downregulated links were upregulated, they would generally indicate worse response. However, we do observe that some of the downregulated interactions in the subnetwork render a collection of targets for other kinds of therapies. In particular, targets of anti-hormone therapy (e.g. ESR1 and AR) are part of the subnetwork boxed in green.

Summing up, our work hypothesis is supported, as follows. By using ExprEssence, some of the most important known mechanisms related to the differences between responders and non-responders could be extracted. In addition, this subnetwork gives rise to new hypotheses with regard to the mechanistic workings of TFAC. One of these mechanisms will be investigated further in the following section. The interactions that are not featured in the main text are discussed in the Supplement (Supplementary Text).

### The role of MELK and MYBL2 as targets for TFAC therapy

In Figure 1 (bottom right), we identified the stimulation of MYBL2 (also known as B-MYB) by MELK to be upregulated in responders. At first glance, this finding is contradictory to the general association of high MYBL2 and MELK expression levels with aggressive tumor growth and poor outcome in breast cancer and other tumors [25]-[27]. However, similar to the cell cycle proteins boxed in red in Figure 1, MELK, MYBL2 and their interaction may be a target for TFAC therapy.

More specifically, MELK is expressed in several developing tissues, but it is also found in breast tumor-initiating cells, and is required for mammary tumor growth in vivo [28]. Moreover, the proto-oncogene MYBL2 is known to allow cells to override growth inhibitory signals and is essential for S-phase entry [29]–[31]. The BioGraph Database [32] and the Comparative Toxicogenomics Database (CTD, [33]) suggest that MELK is related to susceptibility to TFAC therapy. Both databases refer to analyses performed by Hess et al., where MELK has been observed to be significantly upregulated in responders to TFAC therapy [34], although no *direct* effects of paclitaxel on MELK were reported there. MELK is known to stimulate MYBL2 based on observations by Nakano et al., who found a downregulation of MYBL2 to be induced by MELK knockdown [35]. Thus, we decided to investigate the underlying stimulation, and its possible inhibition by paclitaxel, in more depth. In the literature we found indications that paclitaxel inhibits MELK via E2F transcription factors, and that MELK stimulates MYBL2 via ZPR9, implying that paclitaxel inhibits MYBL2 indirectly via MELK.

More specifically, paclitaxel has been shown to induce the cyclin inhibitor p21*^WAF^* ^1^ in MCF-7 breast cancer cells [36], which leads to lower Cdk2 activity [37], resulting in less phosphorylation of the pocket proteins p107/p130 and persistent association of E2F transcription factors with p107/p130 [38]. Verlinden et al. found the MELK gene to carry E2F responsive elements in its promoter region [39]. Hence, paclitaxel-induced complexation of E2F transcription factors could lead to a downregulation of MELK gene expression. This could trigger less MYBL2 expression, since MELK has been shown to phosphorylate the zinc-finger-like protein ZPR9 [40], which in turn enhances transcriptional activity of MYBL2 [35], [41]. Moreover, Nakano et al. suggested that both ZPR9 and MYBL2 are transcriptionally regulated by MELK [35]. (Since ZPR9 is not represented in the data we used, it could not become a member of our E40 network.)

According to Calvisi et al. [42], low expression of MYBL2 is beneficial for chemotherapy response. More specifically, Calvisi et al. investigated hepatocellular carcinoma (HCC) cell lines with wildtype and mutated p53 and found MYBL2-inhibited HCC cells to be associated with reduced proliferation, increased DNA damage, and induction of apoptosis irrespective of p53 status. A p53 mutant status was correlated with higher levels of MYBL2 and advanced tumor stage of human breast cancer [43]. However, especially HCC cells with mutated p53, which are not able to arrest in the G_1_ phase and therefore enter into mitosis with DNA heavily damaged by doxorubicin, show higher rates of apoptosis than p53 wildtype HCC cells. Therefore, Calvisi et al. concluded that MYBL2 inhibition could represent a valuable adjuvant for doxorubicin treatment against human hepatocellular carcinoma especially with mutated p53.

Taken together, a hypothesis resulting from our ExprEssence analysis is that paclitaxel plays an important role as a co-player of doxorubicin by repressing MELK expression, which in turn attenuates MYBL2 expression and hence allows for more efficient effects of doxorubicin. We investigated this hypothesis experimentally using several breast cancer cell lines as described below.

### Experimental investigation of chemotherapy effects on MELK and MYBL2

Four different epithelial breast cell lines were chosen to compare effects imputed to breast cancer subtype (Table 2). As a non-tumorigenic/normal breast-like control the cell line MCF-10A was selected. The cell line MCF-7 represents the most prevalent and most common breast cancer subtype (luminal, estrogen receptor (ER) and progesterone receptor (PR) positive). The highly invasive cell line BT-20 was used as a model for the triple negative type because neither ER, PR nor human epidermal growth factor receptor 2 (HER2) expression is observed in BT-20. As HER2 positive cell type the cell line SKBR3 was used. Prior treatment with chemotherapeutic agents, all cells were adapted to phenol red free medium with charcoal treated serum to avoid cross stimulation with endogenous hormones like 17*β*-estradiol. Final concentrations of paclitaxel and doxorubicin were selected on the basis of published IC50 values for both substances [44].

**Table 2:** Selected breast cancer subtypes with their most common marker profile, their overall prevalence and a representative human cell line with these molecular features.

Subtype	Markers	Prevalence	Cell line
Luminal	ER^+^ and/or PR^+^, HER2^−^, low Ki67	42–59%	MCF-7
Triple negative	ER^−^, PR^−^, HER2^−^, cytokeratin 5/6^+^	14–20%	BT-20
HER2^+^	ER^−^, PR^−^, HER2^−^	7–12%	SKBR3
Non-tumorigenic/basal-like/ normal breast-like	ER^+/−^ and/or PR^+/−^, HER2^−^	–	MCF-10A

This table was compiled from different sources [45]–[48]. ER: Estrogen receptor; PR: Progesterone receptor; HER2: human epidermal growth factor receptor 2; +: positive; −: negative.

For our experimental setup, we tested paclitaxel alone for 48 h (T), doxorubicin alone for 48 h (A), a combination (T + A) (48 h) and successive treatment so that paclitaxel was first given for 24 h and thereafter doxorubicin was added (T (24 h), A (24 h)).

**Figure 2:**
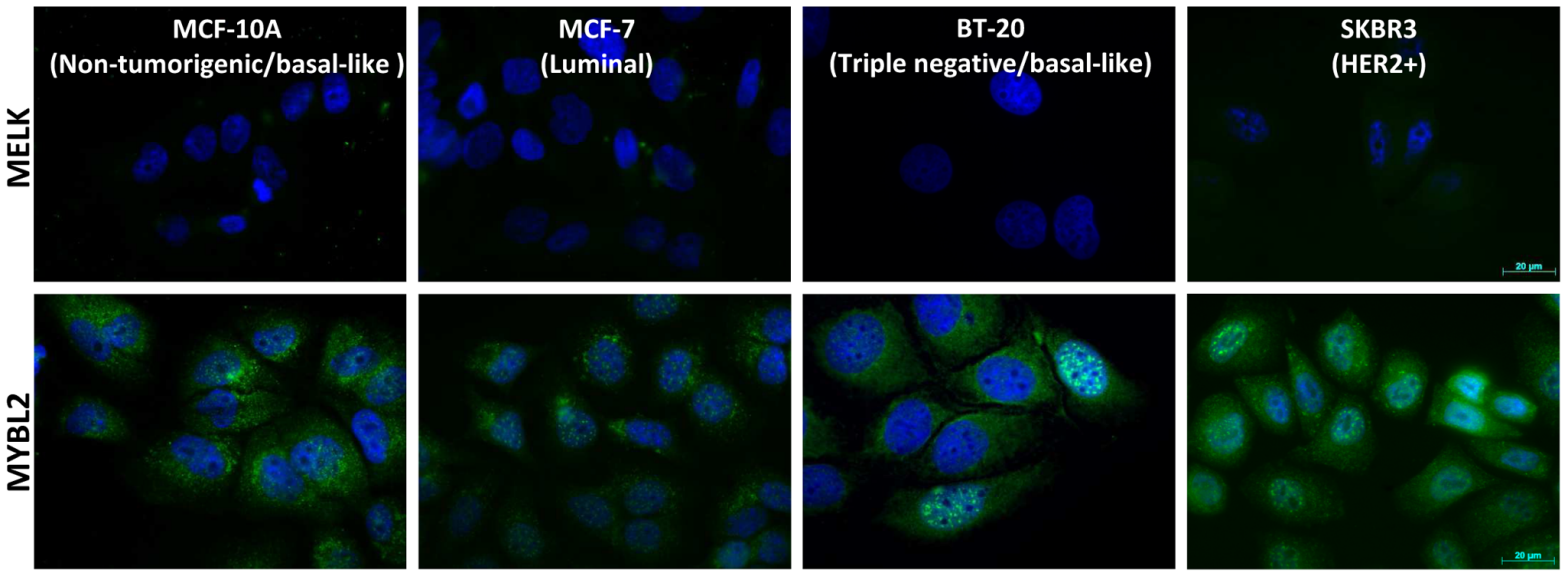
Expression levels of MELK and MYBL2 protein in the non-tumorigenic cell line MCF-10A in contrast to the breast cancer cell lines MCF-7, BT-20 and SKBR3 detected by immunofluorescence. Note that MELK protein levels were below detection threshold while MYBL2 protein was abundant in all cell lines. The strongest MYBL2 signal was reached in the cell line SKBR3. MELK and MYBL2 protein: green; cell nuclei: blue.

**Figure 3:**
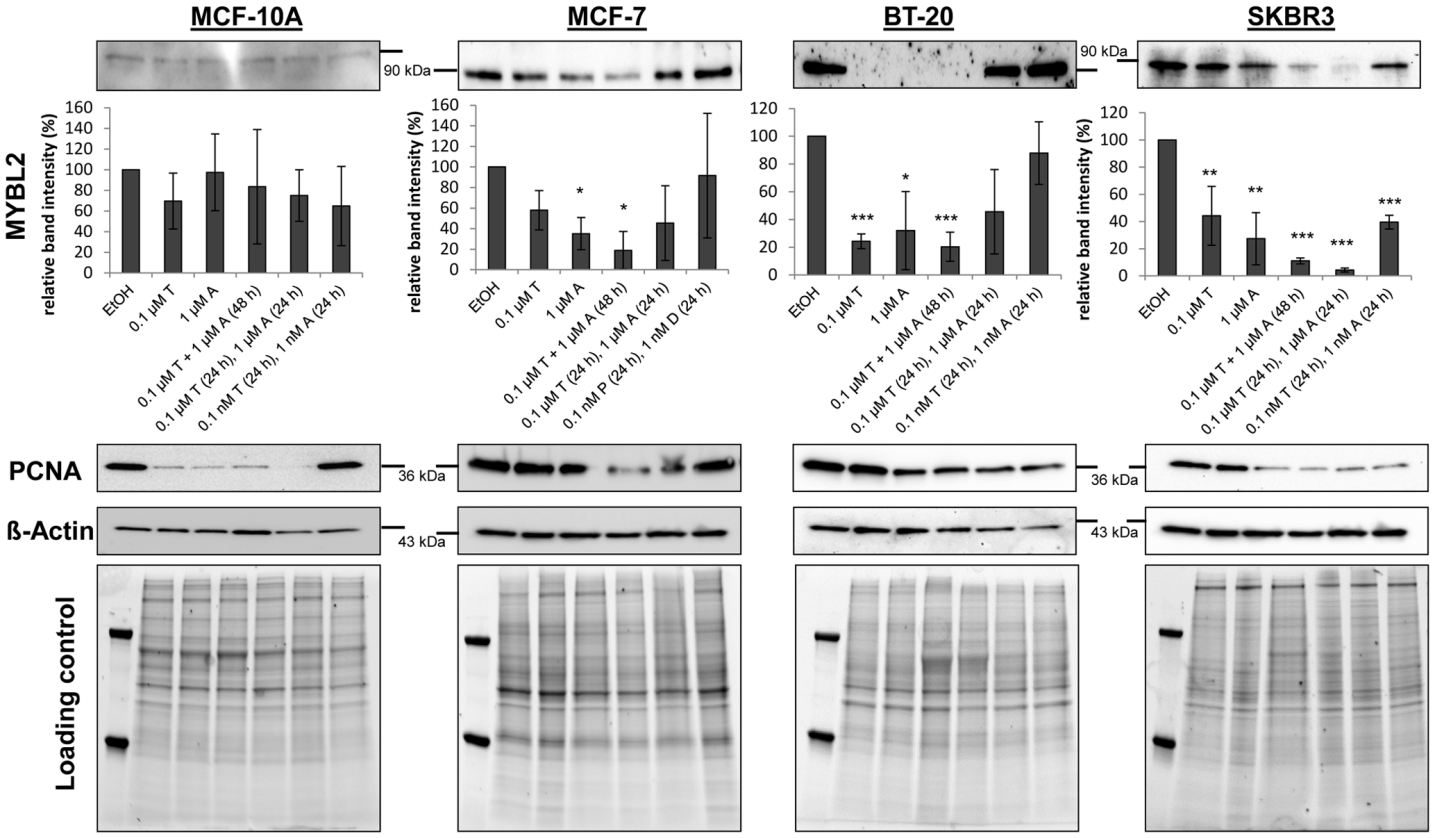
Expression analysis of MYBL2 protein after treatment with paclitaxel (Taxol, T) and doxorubicin (Adriamycin, A) in several cell lines by Western blotting (non-tumorigenic cell line MCF-10A and breast cancer cell lines MCF-7, BT-20 and SKBR3). Single treatment with T or A for 48(T (48 h); A (48 h)), combined treatment for 48 h (T + A (48 h)) or successive treatment for each for 24 h (T (24 h), A (24 h) was applied. Quantification of western blotting results was carried out with individual passaged cells for at least three times. Representative western blots were displayed on top of the graphs. Proliferative alterations were detected against Proliferating Cell Nuclear Antigen (PCNA). Loading controls were labeling of the house keeping protein *β*-actin and stain-free imaging of the SDS-PAGEs prior blotting procedure. Mean ± SD values (n = 3). * : *p*<0.05; ** : *p*<0.01; * * * : *p*<0.001 as compared to control treatment (unpaired t test).

Probably due to very low levels of MELK protein in the breast cancer cell lines used in this study, we were not able to detect MELK using immunofluorescence and western blotting (Figure 2). This reflects that primarily tumor-initiating cells or stroma cells, which are not represented by the used cell cultures, express MELK [28]. Accordingly, MELK gene expression of the specimens investigated in this study indicates that they originate from freshly diagnosed breast cancer tissue which may, besides breast cancer cells, contain also tumor-initiating and stroma cells. In contrast, we observed high levels of MYBL2 especially in the breast cancer cell lines (Figures 2, 3) and a decrease of MYBL2 protein levels after application of T and A both individually and in combination could be verified by western blotting, see below.

The various treatment combinations showed the direct influence of the single agents and also the combined effects on the protein expression level of MYBL2. In the left panel of Figure 3, the protein expression level of MYBL2 in the non-tumorigenic cell line MCF-10A after treatment with chemotherapeutic agents in comparison with vehicle control is given. The representative blot as well as the densitometric statistics of the three individual replicates shows that MYBL2 is expressed with no significant alterations in the non-tumorigenic cell line MCF-10A after treatment with the chemotherapeutics. As a marker for proliferative behavior, the Proliferating Cell Nuclear Antigen, commonly known as PCNA, was detected on the same blots. PCNA expression was significantly reduced after treatment with T and A alone as well as with the combined treatments. Only the lowest concentrations (0.1 nM T+1 nM A) caused no proliferative alterations in comparison with control. This result reflects the strong inhibitory effect of T and A on proliferation of dividing cell populations by either stabilizing microtubules or by intercalating DNA. As loading control, the counter labeling with *β*-actin as a housekeeping protein and also the stain-free imaging of the SDS-PAGE separations for visual monitoring of the loaded total protein contents were utilized.

Further, the western blotting experiments of the breast cancer cell lines (MCF-7, BT-20, SKBR3) demonstrated that these displayed significantly stronger expression levels of MYBL2 in the untreated state compared to the non-tumorigenic control (MCF-10A). On each blot, 10 µg total protein was transferred, making the expression levels of the cell lines comparable. The high expression levels of MYBL2 in the cancer cell lines render them as potential targets for treatment with the chemotherapeutic agents. In contrast to the non-tumorigenic cell line, MYBL2 expression levels of the breast cancer cell lines showed a distinct response to treatment with the chemotherapeutic agents (Figure 3). For MCF-7, a significant reduction of MYBL2 expression (by ∼80%) after treatment with the simultaneously given agents (T + A (48 h)) was observed. Furthermore, the exposure to 1 µM A alone revealed a significant reduction of MYBL2 expression. The triple negative breast cancer cell line, BT-20, and the HER2 positive one, SKBR3, displayed a strong response after the simultaneous treatment with both chemotherapeutics (Figure 3). The expression levels of MYBL2 in BT-20 cells decreased up to approximately 80%. For SKBR3 cells, a reduction of 95% for MYBL2 protein was reached. But in contrast to MCF-7, the single agents also influenced the protein contents of BT-20 and SKBR3. BT-20 cells showed a strong downregulation of MYBL2 protein after 0.1 µM T or 1 µM A exposure, similar to their combined application. The successive exposure with chemotherapeutic agents did not further enhance the protein repression in BT-20 cells. The influence on SKBR3 cells turned out to be somewhat different. Although the single chemotherapeutics paclitaxel and doxorubicin decreased MYBL2 expression significantly, the highest downregulation was reached after combined or successive treatment.

In conclusion, the combined exposure of paclitaxel and doxorubicin (T + A (48 h)) revealed the strongest response on MYBL2 repression in the breast cancer cell lines (MCF-7, BT-20, SKBR3) while non-tumorigenic control cells (MCF-10A) were not affected. All three tested breast cancer cell lines were sensitive for the combined treatment of both chemotherapeutic adjuvants, as reflected by decreased PCNA expression, except for the lowest treatment conditions in MCF-7 cells. However, SKBR3 and not BT-20 cells showed the most sensitive response to the combined chemotherapeutic treatment with paclitaxel and doxorubicin in concordance with repression of MYBL2 contents. Therefore, further investigations should be performed on MYBL2 as a marker for TFAC chemotherapy response in breast cancer cells, in particular in HER2-positive cells.

Currently, TFAC therapy is preferably used for triple-negative breast tumors [20]. Therefore, we also analyzed the cytotoxic potential of the combined treatment of paclitaxel and doxorubicin in the triple-negative cell line BT-20 in comparison to the non-tumorigenic control (MCF-10A) (Figure S6). Towards this end, we decided to use three independent cytotoxic measurement methods (MTS assay, Live-Dead-assay and cell cycle measurements for proliferation and apoptosis determination). The MTS assay reflects the influence on the metabolic viability of the cells, while the live-dead-test directly provides information about the induction of apoptosis. Viability was significantly lowered after treatment with A alone and in combination with T in both cell lines (Figure 3). In contrast, the live-dead staining revealed a significant higher level of apoptotic BT-20 cells after exposure to 1 µM A and the combined treatment with T (Figure S6 (B), (D)).

Finally, we analyzed the cell cycle phases by flow cytometry since paclitaxel stabilizes microtubules and induces a G2-phase arrest. As expected, T alone induced an arrest in the G2-phase, leading to higher proliferation rates (G2/M + S phase) in both cell lines. Though the increase in proliferation rate was significant for both MCF-10A and BT-20 cells, the absolute change was only marginal for BT-20 cells (Figure S6 (C)). Treatment with A or the combined exposure of both agents showed a significant decrease of the proliferative phases in BT-20 cells, while MCF-10A proliferation was stimulated. This effect on MCF-10A cells is not unusual, since, in an epithelial tissue, apoptosis is often compensated for by increased proliferation rates to maintain the tissue structure. These three cytotoxic assays confirm the postulated effects of T and A on the BT-20 cell line, a representative of the triple-negative breast cancer subtype. Furthermore, these results validate the MYBL2 western blotting experiments, demonstrating that the combined exposure to T and A leads to the strongest effects.

We can summarize that high expression levels of MYBL2 are associated with response to TFAC treatment, which should be verified in further experiments by the investigation of human tissue material. The results of the bioinformatics analysis are consistent with cell biological results concerning the downregulation of MYBL2 protein induced by TFAC treatment, rendering MYBL2 as a potential breast cancer marker for a successful TFAC therapy, with a putative mechanistic connection to MELK.

## Conclusions

We applied ExprEssence, a software tool for the extraction of differentially regulated interactions from an interaction network, to preoperative breast cancer chemotherapy response and compared the resulting subnetwork to the results of two other subnetwork-identifying methods, OptDis and KeyPathwayMiner. Performing an IPA Functional Enrichment Analysis, we demonstrated that the genes exhhibited by ExprEssence are more closely related to the mode of functioning of TFAC therapy, compared to the other methods.

Just like the other methods OptDis and KeyPathwayMiner, our method relies on a network of gene/protein interactions onto which gene expression data is mapped. A disadvantage of starting with a known network is that we are not able to discover novel interactions and hence false negatives may arise. Also, gene expression data may not reflect post-translational modifications such as phosphorylations. Furthermore, using gene expression data being collected over a period of many years most likely involves specimen retrieval by several people and may also comprise changes of technical protocols, both possibly leading to biased data which cannot be compensated for. Nevertheless, we can generate valuable hypotheses based on highlighting some interactions as particularly relevant. These may be false positives, since network interactions are context-dependent events, and gene expression data may give false evidence in cases where changes of gene expression are irrelevant. Thus, the highlighted interactions we found may not give a complete picture, and they need to be validated experimentally.

In the case study presented here, besides identifying interactions already known to be related to TFAC therapy response, we proposed a putative response-related mechanism via MELK and MYBL2, which has not been taken into account yet for assessment of response. We performed experiments with cell lines representing various breast cancer subtypes to test our hypothesis that paclitaxel acts synergistically with doxorubicin via suppression of MELK, which in turn attenuates MYBL2 gene expression, known to be advantageous for chemotherapy response. Though, probably due to low amounts, we were not able to detect MELK protein, we could demonstrate attenuated MYBL2 protein levels in chemotherapy treated cells and a synergism of paclitaxel and doxorubicin. Concludingly, with the stimulation of MYBL2 by MELK, we identified an interaction of potential relevance for decision-making on TFAC therapy.

## Supporting Information

Figure S1 **KeyPathwayMiner subnetwork 1.** The green nodes represent genes that are active (i.e. genes that are differentially expressed between responders and non-responders), exception nodes (genes not being differentially expressed) are drawn red. The number of exception nodes is one parameter of KeyPathwayMiner - here it was set to 8.
(EPS)

Figure S2 **KeyPathwayMiner subnetwork 2. The meaning of red and green colors is explained in Figure S1.**
(EPS)

Figure S3 **KeyPathwayMiner subnetwork 3. The meaning of red and green colors is explained in Figure S1.**
(EPS)

Figure S4 **KeyPathwayMiner subnetwork 4. The meaning of red and green colors is explained in Figure S1.**
(EPS)

Figure S5 **KeyPathwayMiner subnetwork 5. The meaning of red and green colors is explained in Figure S1.**
(EPS)

Figure S6 **Cytotoxic activity on non-tumorigenic control cell line MCF-10A (black bar) and triple negative breast cancer cell line BT-20 (grey bar) after treatment with paclitaxel (T) and doxorubicin (A) was calculated by three individual assays: MTS (A), Live-Dead (B, D) and Cell cycle analysis (C). In each measurement the control treatment with 0.1% EtOH was set to 100% to validate the results after exposure to the compounds.** All measurements were repeated at a minimum of three replicates. Fluorescence pictures of live (green) and dead (red) stained cells were taken with a fluorescence microscope (Axio Scope. A1, Carl Zeiss, Germany). Mean ± SD values (*n* = 3). *:*p*<0.05; **:*p*<0.01; ***:*p*<0.001 as compared to control treatment (unpaired t test).
(TIF)

Table S1 **Full gene names for the gene symbols in** **Figure 1** **.**
(PDF)

Table S2 **Genes of the top 5 KeyPathwayMiner subnetworks.**
(PDF)

Table S3 **Top 25 Biological Functions terms of the IPA analysis for the top 5 KeyPathwayMiner subnetworks.**
(PDF)

Table S4 **Top 25 terms of Ingenuity Functional Enrichment Analysis for E40 gene set including associated genes and p-values (corrected for multiple testing using BenjaminiHochberg correction).**
(PDF)

Table S5 **Top 25 terms of Ingenuity Functional Enrichment Analysis for O39 gene set including associated genes and p-values (corrected for multiple testing using BenjaminiHochberg correction).**
(PDF)

Text S1 (PDF)
