# Supplementary figures and images for: Differential Network Analysis Applied to Preoperative Breast Cancer Chemotherapy Response
Source: PLoS One. 2013 Dec 9;8(12):e81784. doi: 10.1371/journal.pone.0081784 (PMC3857210; doi:10.1371/journal.pone.0081784)

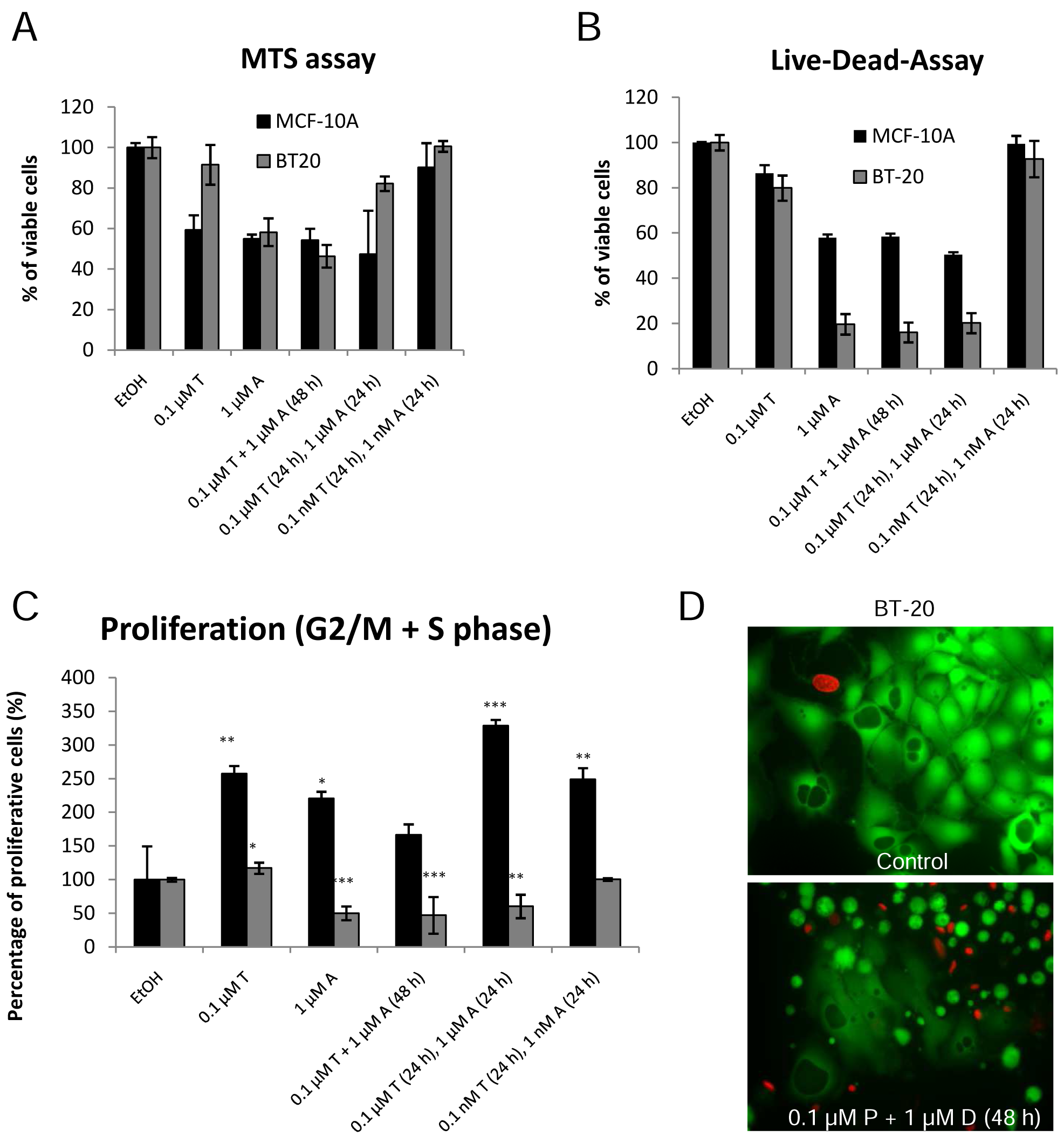

Supplement: Figure S6 — Cytotoxic activity on non-tumorigenic control cell line MCF-10A (black bar) and triple negative breast cancer cell line BT-20 (grey bar) after treatment with paclitaxel (T) and doxorubicin (A) was calculated by three individual assays: MTS (A), Live-Dead (B, D) and Cell cycle analysis (C). In each measurement the control treatment with 0.1% EtOH was set to 100% to validate the results after exposure to the compounds. All measurements were repeated at a minimum of three replicates. Fluorescence pictures of live (green) and dead (red) stained cells were taken with a fluorescence microscope (Axio Scope. A1, Carl Zeiss, Germany). Mean ± SD values (n = 3). *:p<0.05; **:p<0.01; ***:p<0.001 as compared to control treatment (unpaired t test). (TIF) [file pone.0081784.s006.tif]
