# Supplementary material for: Differential Network Analysis Applied to Preoperative Breast Cancer Chemotherapy Response
Source: PLoS One. 2013 Dec 9;8(12):e81784. doi: 10.1371/journal.pone.0081784 (PMC3857210; doi:10.1371/journal.pone.0081784)
Supplement: Table S1 — Full gene names for the gene symbols in Figure 1 . (PDF) [file pone.0081784.s007.pdf]

Gene Symbol	Gene Name
AGR2	anterior gradient 2 homolog (Xenopus laevis)
AR	androgen receptor
CCND1	cyclin D1
CDC20	cell division cycle 20
CDCA8	cell division cycle associated 8
CENPE	centromere protein E, 312kDa
CENPN	centromere protein N
COL1A2	collagen, type I, alpha 2
CPB1	carboxypeptidase B1 (tissue)
ESR1	estrogen receptor 1
FOXA1	forkhead box A1
GATA3	GATA binding protein 3
GFRA1	GDNF family receptor alpha 1
HJURP	Holliday junction recognition protein
IRS1	insulin receptor substrate 1
KIF18A	kinesin family member 18A
KIF20A	kinesin family member 20A
KIFC1	kinesin family member C1
KRT15	keratin 15
KRT16	keratin 16
KRT17	keratin 17
KRT7	keratin 7
LYPD3	LY6/PLAUR domain containing 3
MELK	maternal embryonic leucine zipper kinase
MID1	midline 1 (Opitz/BBB syndrome)
MYB	v-myb myeloblastosis viral oncogene homolog (avian)
MYBL2	v-myb myeloblastosis viral oncogene homolog (avian)-like 2
NDC80	NDC80 kinetochore complex component
PGR	progesterone receptor
PLAT	plasminogen activator, tissue
RUVBL2	RuvB-like 2 (E. coli)
S100A1	S100 calcium binding protein A1
S100A7	S100 calcium binding protein A7
SERPINA3	serpin peptidase inhibitor, clade A (alpha-1 antiproteinase, antitrypsin), member 3
SERPINA5	serpin peptidase inhibitor, clade A (alpha-1 antiproteinase, antitrypsin), member 5
SERPINA6	serpin peptidase inhibitor, clade A (alpha-1 antiproteinase, antitrypsin), member 6

Table S1. Full gene names for the gene symbols in Figure 1.
