# Supplementary material for: Differential Network Analysis Applied to Preoperative Breast Cancer Chemotherapy Response
Source: PLoS One. 2013 Dec 9;8(12):e81784. doi: 10.1371/journal.pone.0081784 (PMC3857210; doi:10.1371/journal.pone.0081784)
Supplement: Table S2 — Genes of the top 5 KeyPathwayMiner subnetworks. (PDF) [file pone.0081784.s008.pdf]

KPM1	KPM2	KPM3	KPM4	KPM5
CCND1	AKT1	AKT1	AKT1	AKT1
DGKI	CCND1	CCND1	ANXA7	CCND1
EFNB2	DGKI	DGKI	CCND1	DEPDC6
EN1	EN1	EFNB1	DGKI	DGKI
EPHB3	EPHB3	EN1	DOCK1	EN1
ERBB2	ERBB2	EPHB3	EN1	EPHB3
FOXC1	FOXC1	ERBB2	ERBB2	ERBB2
FZD1	FZD7	FOXC1	FOXC1	FOXC1
FZD7	GATA3	FZD7	FZD7	FZD7
GATA3	GDNF	GATA3	GATA3	GATA3
GDNF	GRB7	GRB7	GRB7	GDNF
GRB7	HNF4A	ITGA6	HNRNPA2B1	GRB7
IGFBP4	IGF1	KLF5	ITGA6	HNF4A
ITGA6	IGFBP4	LIPC	KLF5	ITGA6
KLF5	ITGA6	LPIN1	LIPC	KLF5
LPIN1	KLF5	MLST8	LPIN1	LPIN1
MAX	LPIN1	MSX2	MSX2	MSX2
MSX2	MSX2	NRTN	NRTN	NRTN
NDRG2	NRTN	PPARG	PPARG	PLCG1
NRTN	PLCG1	RASGRP3	RASGRP3	PPARG
PLCG1	PPARG	RET	RET	RASGRP3
PPARG	RASGRP3	RRAGC	RXRB	RRAGC
RASGRP3	RXRB	RXRB	SF3A3	RXRB
RXRB	SHH	SHH	SHH	SHH
SHH	SOX10	SOX10	SOX10	SOX10
SOX10	TRIB3	TRIB3	TRIB3	TRIB3
WNT1	WNT1	WNT1	WNT1	WNT1
ZIC1	ZIC1	ZIC1	ZIC1	ZIC1

Table S2. Genes of the top 5 KeyPathwayMiner subnetworks.
