# Supplementary material for: Differential Network Analysis Applied to Preoperative Breast Cancer Chemotherapy Response
Source: PLoS One. 2013 Dec 9;8(12):e81784. doi: 10.1371/journal.pone.0081784 (PMC3857210; doi:10.1371/journal.pone.0081784)
Supplement: Table S3 — Top 25 Biological Functions terms of the IPA analysis for the top 5 KeyPathwayMiner subnetworks. (PDF) [file pone.0081784.s009.pdf]

| KPM1 | KPM2 | KPM3 | KPM4 | KPM5 |
|---|---|---|---|---|
| Differentiation of cells | Differentiation of cells | Differentiation of cells | Differentiation of cells | Differentiation of cells |
| Proliferation of neuronal cells | Proliferation of neuronal cells | Migration of neural crest cells | Proliferation of neuronal cells | Proliferation of neuronal cells |
| Migration of neural crest cells | Transactivation of RNA | Proliferation of neuronal cells | Expression of RNA | Development of central nervous system |
| Cell movement | Development of central nervous system | Morphology of nervous system | Transcription of RNA | Expression of RNA |
| Development of central nervous system | Expression of RNA | Development of thymus gland | Development of tumor | Transcription of RNA |
| Migration of cells | Transcription of RNA | Transactivation of RNA | Migration of neural crest cells | Transcription of DNA |
| Transactivation of RNA | Transcription of DNA | Development of central nervous system | Transactivation of RNA | Migration of neural crest cells |
| Expression of RNA | Differentiation of adipocytes | Expression of RNA | Hypoplasia | Transactivation of RNA |
| Transcription of RNA | Morphology of nervous system | Morphology of brain | Differentiation of adipocytes | Differentiation of adipocytes |
| Proliferation of cells | Activation of DNA endogenous promoter | Morphology of brain | Quantity of tumor | Morphology of nervous system |
| Development of autonomic nervous system | Development of sensory organ | Development of lymphatic system component | Development of autonomic nervous system | Development of autonomic nervous system |
| Transcription of DNA | Development of tumor | Transcription of RNA | Transcription of DNA | Activation of DNA endogenous promoter |
| Apoptosis | Cell movement | Hypoplasia | Differentiation of tumor cells | Differentiation of tumor cells |
| Abnormal morphology of embryonic tissue | Proliferation of embryonic cells | Morphology of head | Triple-negative breast cancer | Development of sensory organ |
| Development of brain | Migration of neural crest cells | Differentiation of adipocytes | Development of central nervous system | Apoptosis |
| Differentiation of muscle cells | Migration of cells | Quantity of tumor | Proliferation of embryonic cells | Development of tumor |
| Development of lymphatic system component | Differentiation of muscle cells | Development of autonomic nervous system | Proliferation of cells | Development of thymus gland |
| Morphology of head | Apoptosis | Transcription of DNA | Morphology of brain | Development of brain |
| Morphology of nervous system | Development of brain | Development of sensory organ | Differentiation of fibroblast cell lines | Proliferation of embryonic cells |
| Activation of DNA endogenous promoter | Migration of embryonic cells | Triple-negative breast cancer | Differentiation of muscle cells | Induction of tumor |
| Development of cerebellum | Survival of dopaminergic neurons | Development of tumor | Morphology of nervous system | Morphology of brain |
| Development of body axis | Morphology of brain | Cell viability of neurons | Development of cerebellum | Differentiation of fibroblast cell lines |
| Abnormal morphology of endolymphatic duct | Development of lymphatic system component | Proliferation of embryonic cells | Cell movement | Differentiation of muscle cells |
| Proliferation of epithelial cells | Proliferation of cells | Differentiation of fibroblast cell lines | Regulation of cells | Development of lymphatic system component |
| Cell death | Hypoplasia | Differentiation of muscle cells | Proliferation of endocrine cell lines | Hypoplasia |

**Table S3.** Top 25 Biological Functions terms of the IPA analysis for the top 5 KeyPathwayMiner subnetworks.
