# Supplementary material for: Differential Network Analysis Applied to Preoperative Breast Cancer Chemotherapy Response
Source: PLoS One. 2013 Dec 9;8(12):e81784. doi: 10.1371/journal.pone.0081784 (PMC3857210; doi:10.1371/journal.pone.0081784)
Supplement: Table S4 — Top 25 terms of Ingenuity Functional Enrichment Analysis for E40 gene set including associated genes and p-values (corrected for multiple testing using BenjaminiHochberg correction). (PDF) [file pone.0081784.s010.pdf]

| Functions Annotation | p-value | Molecules |
|---|---|---|
| Carcinoma | 2.00E-07 | AGR2, AR, CCND1, CDC20, CDCA8, COL1A2, ESR1, FOXA1, GATA3, GFRA1, IRS1, KIF20A, KIFC1, KRT16, KRT7, MELK, MYB, MYBL2, PGR, PLAT, S100A1, SERPINA3, SERPINA5, SERPINA6, TFF1, TFF3, ZEB1 |
| Cell cycle progression | 2.00E-07 | AR, CCND1, CDC20, CDCA8, CENPE, ESR1, FOXA1, GATA3, HJURP, IRS1, KIFC1, MELK, MYB, MYBL2, NDC80, PGR, SERPINA5, ZEB1 |
| Chromosomal congression of chromosomes | 1.58E-06 | CENPE, KIF18A, KIFC1, NDC80 |
| Cancer | 1.58E-06 | AGR2, AR, CCND1, CDC20, CDCA8, COL1A2, ESR1, FOXA1, GATA3, GFRA1, IRS1, KIF20A, KIFC1, KRT15, KRT16, KRT17, KRT7, MELK, MYB, MYBL2, NDC80, PGR, PLAT, S100A1, SERPINA3, SERPINA5, SERPINA6, TFF1, TFF3, ZEB1 |
| Carcinoma in breast | 1.58E-06 | AGR2, AR, CCND1, COL1A2, ESR1, GATA3, GFRA1, MYBL2, PGR, SERPINA5, TFF1 |
| Breast cancer | 1.58E-06 | AGR2, AR, CCND1, CDC20, COL1A2, ESR1, FOXA1, GATA3, GFRA1, KRT15, KRT17, MYB, MYBL2, PGR, SERPINA5, TFF1 |
| Digestive organ tumor | 1.89E-05 | AR, CCND1, CDC20, CDCA8, COL1A2, ESR1, FOXA1, IRS1, KIF20A, KIFC1, KRT7, MELK, MYB, PGR, PLAT, SERPINA3, SERPINA6, TFF1, TFF3 |
| Proliferation of cells | 3.49E-05 | AGR2, AR, CCND1, CDC20, CDCA8, COL1A2, ESR1, FOXA1, GATA3, GFRA1, IRS1, KIF20A, KRT16, KRT17, MELK, MYB, MYBL2, PGR, PLAT, S100A1, S100A7, SERPINA5, SERPINH1, TFF1, TFF3, ZEB1 |
| Proliferation of tumor cells | 6.25E-05 | AR, CCND1, ESR1, GFRA1, IRS1, MYB, MYBL2, PLAT, S100A7, TFF3 |
| Plaque psoriasis | 6.25E-05 | CCND1, GATA3, KRT15, KRT16, KRT17, S100A7 |
| Cell movement | 7.72E-05 | AGR2, AR, CCND1, ESR1, FOXA1, GATA3, GFRA1, IRS1, KRT16, LYPD3, MYB, PLAT, S100A1, S100A7, SERPINA3, SERPINA5, TFF1, TFF3, ZEB1 |
| Proliferation of breast cancer cell lines | 1.00E-04 | AR, CCND1, ESR1, IRS1, MYB, PGR, S100A1, S100A7, ZEB1 |
| Amenorrhea | 2.46E-04 | AR, PGR, SERPINA6 |
| Cell cycle progression of tumor cell lines | 2.65E-04 | AR, CCND1, ESR1, FOXA1, MELK, PGR, ZEB1 |
| Proliferation of cancer cells | 2.98E-04 | ESR1, GFRA1, IRS1, MYB, MYBL2, PLAT, S100A7, TFF3 |
| Hyperplasia | 3.32E-04 | AR, CCND1, ESR1, GATA3, IRS1, KRT16, KRT17, MYB, PGR, S100A7 |
| Mitosis | 3.98E-04 | AR, CCND1, CDC20, CDCA8, CENPE, IRS1, KIFC1, MYBL2, NDC80 |
| Skin development | 4.33E-04 | CCND1, COL1A2, GFRA1, KRT15, KRT16, KRT17, S100A7 |
| Development of epidermis | 4.92E-04 | CCND1, GFRA1, KRT15, KRT16, KRT17, S100A7 |
| Cell cycle progression of breast cancer cell lines | 5.04E-04 | CCND1, ESR1, MELK, PGR |
| Cell viability of endometrial cells | 5.06E-04 | ESR1, PGR |
| Cloning of fibroblast cell lines | 5.06E-04 | CCND1, MYBL2 |
| Infanticide | 5.06E-04 | ESR1, PGR |
| Mammary gland development | 5.09E-04 | AR, CCND1, ESR1, GATA3, IRS1, PGR |
| Carcinoma in situ | 5.09E-04 | AR, ESR1, GATA3, KRT16, KRT7, TFF3 |

**Table S4.** Top 25 terms of Ingenuity Functional Enrichment Analysis for E40 gene set including associated genes and p-values (corrected for multiple testing using BenjaminiHochberg correction).
