# Supplementary material for: Differential Network Analysis Applied to Preoperative Breast Cancer Chemotherapy Response
Source: PLoS One. 2013 Dec 9;8(12):e81784. doi: 10.1371/journal.pone.0081784 (PMC3857210; doi:10.1371/journal.pone.0081784)
Supplement: Table S5 — Top 25 terms of Ingenuity Functional Enrichment Analysis for O39 gene set including associated genes and p-values (corrected for multiple testing using BenjaminiHochberg correction). (PDF) [file pone.0081784.s011.pdf]

Functions Annotation	p-value	Molecules
Transactivation	2.31E-12	AR, EP300, ESR1, GADD45G, GATA3, IGFBP4, MAPK3, MDM2, MED1, MED31, NCOA3, PRKACA, RARA, RET, SMAD3, SRC, STUB1, UBE2I, ZBTB16
Cell movement	4.89E-11	AGR2, AR, C4B (includes others), CST3, ESR1, EVL, GATA3, GFRA1, IGF1R, IGF2, IGFBP4, IL6ST, IRS1, MAPK3, MED1, NCOA3, PRKACA, RABEP1, RARA, RET, SEMA6A, SHC1, SMAD3, SRC, TSC2, UBE2I, ZBTB16
Cell cycle progression	2.37E-10	AR, EP300, ESR1, GADD45G, GATA3, IGF1R, IGF2, IRS1, MAPK3, MDM2, NCOA3, PRKACA, RARA, RET, SHC1, SMAD3, SRC, TSC2, UBE2I, YWHAB, ZBTB16
Migration of cells	2.44E-10	AR, C4B (includes others), CST3, ESR1, EVL, GATA3, GFRA1, IGF1R, IGF2, IGFBP4, IL6ST, IRS1, MAPK3, NCOA3, PRKACA, RABEP1, RARA, RET, SEMA6A, SHC1, SMAD3, SRC, TSC2, UBE2I, ZBTB16
Proliferation of tumor cell lines	1.96E-09	AR, EP300, ESR1, GADD45G, IGF1R, IGF2, IGFBP4, IL6ST, IRS1, MAPK3, MDM2, MED1, NCOA3, PRKACA, RARA, RET, SHC1, SMAD3, SRC, STUB1, UBE2I, ZBTB16
Apoptosis of tumor cell lines	3.95E-09	AR, EP300, ESR1, GADD45G, IGF1R, IGF2, IGFBP4, IL6ST, MAPK3, MDM2, NCOA3, PRKACA, RARA, RET, SHC1, SMAD3, SRC, STUB1, TSC2, ZBTB16
Proliferation of connective tissue cells	5.15E-09	AR, ESR1, IGF1R, IGF2, IGFBP4, IL6ST, IRS1, MAPK3, MDM2, MED1, RARA, RET, SMAD3, SRC, TSC2
Proliferation of cells	5.15E-09	AGR2, AR, ASH2L, CALM1 (includes others), CST3, EP300, ESR1, GADD45G, GATA3, GFRA1, IGF1R, IGF2, IGFBP4, IL6ST, IRS1, MAPK3, MDM2, MED1, NCOA3, PRKACA, RABEP1, RARA, RET, SEMA6A, SHC1, SMAD3, SRC, STUB1, TSC2, UBE2I, ZBTB16
Necrosis	5.15E-09	AGR2, AR, CST3, EP300, ESR1, GADD45G, GATA3, GFRA1, IGF1R, IGF2, IGFBP4, IL6ST, IRS1, MAPK3, MDM2, MED1, NCOA3, PRKACA, RARA, RET, SHC1, SMAD3, SRC, STUB1, TSC2, YWHAB, ZBTB16
Hypoplasia	5.77E-09	AR, ESR1, IGF1R, IGF2, IL6ST, MDM2, MED1, RARA, RET, SHC1, SMAD3, SRC, STUB1, TSC2
Proliferation of epithelial cells	5.77E-09	AGR2, AR, EP300, ESR1, IGF1R, IGF2, IGFBP4, MED1, RARA, RET, SMAD3, TSC2, ZBTB16
Transcription	6.11E-09	AP1G2, AR, ASH2L, EP300, ESR1, GADD45G, GATA3, IGF2, IL6ST, MAPK3, MDM2, MED1, NCOA3, PRKACA, RARA, RET, SHC1, SMAD3, SMAD9, SRC, STUB1, UBE2I, YWHAB, ZBTB16
Cell death of tumor cell lines	6.78E-09	AGR2, AR, EP300, ESR1, GADD45G, IGF1R, IGF2, IGFBP4, IL6ST, MAPK3, MDM2, NCOA3, PRKACA, RARA, RET, SHC1, SMAD3, SRC, STUB1, TSC2, ZBTB16
Differentiation of cells	8.30E-09	AR, EP300, ESR1, GADD45G, GATA3, GFRA1, IGF1R, IGF2, IL6ST, IRS1, MAPK3, MDM2, MED1, NCOA3, PRKACA, RARA, RET, SHC1, SMAD3, SMAD9, SRC, TSC2, UBE2I, ZBTB16
Organismal death	1.29E-08	AR, C4B (includes others), CST3, EP300, ESR1, GATA3, IGF1R, IGF2, IL6ST, MAPK3, MDM2, MED1, NCOA3, PRKACA, RARA, RET, SHC1, SMAD3, SMAD9, SRC, STUB1, TSC2
Cell survival	1.29E-08	AR, EP300, ESR1, GATA3, GFRA1, IGF1R, IGF2, IL6ST, IRS1, MAPK3, MDM2, MED1, PRKACA, RARA, RET, SHC1, SMAD3, SRC, TSC2, UBE2I
Synthesis of DNA	1.32E-08	AR, EP300, ESR1, IGF1R, IGF2, IGFBP4, IL6ST, IRS1, MDM2, PRKACA, SHC1, SRC, TSC2
Transcription of RNA	2.48E-08	AR, ASH2L, EP300, ESR1, GADD45G, GATA3, IGF2, IL6ST, MAPK3, MDM2, MED1, NCOA3, PRKACA, RARA, RET, SHC1, SMAD3, SMAD9, SRC, STUB1, UBE2I, YWHAB, ZBTB16
Cell viability	2.62E-08	AR, EP300, ESR1, GATA3, GFRA1, IGF1R, IGF2, IL6ST, MAPK3, MDM2, MED1, PRKACA, RARA, RET, SHC1, SMAD3, SRC, TSC2, UBE2I

Continued on next page

Table S5 – continued from previous page

**Functions Annotation**	**p-value**	**Molecules**
Binding of DNA	2.80E-08	AR, CALM1 (includes others), EP300, ESR1, GATA3, IL6ST, MAPK3, MDM2, MED1, PRKACA, RARA, SMAD3, SRC, YWHAB
Quantity of cells	3.81E-08	AGR2, AR, C4B (includes others), EP300, ESR1, GADD45G, GATA3, GFRA1, IGF1R, IGF2, IL6ST, IRS1, MAPK3, MDM2, MED1, RARA, RET, SHC1, SMAD3, SRC, TSC2, ZBTB16
Development of carcinoma	3.81E-08	CST3, NCOA3, RARA, RET, SMAD3, SRC, TSC2
Proliferation of fibroblasts	4.76E-08	ESR1, IGF1R, IGF2, IGFBP4, IL6ST, MAPK3, MDM2, RARA, SMAD3, SRC, TSC2
Development of tumor	4.76E-08	CST3, ESR1, IGF1R, IL6ST, NCOA3, RARA, RET, SMAD3, SRC, TSC2, ZBTB16
Morphology of embryonic tissue	5.03E-08	AR, EP300, ESR1, GATA3, GFRA1, IL6ST, MDM2, PRKACA, RARA, RET, SHC1, SMAD3, SMAD9, UBE2I

Table S5. Top 25 terms of Ingenuity Functional Enrichment Analysis for O39 gene set including associated genes and p-values (corrected for multiple testing using BenjaminiHochberg correction).
