# Supplementary material for: Differential Network Analysis Applied to Preoperative Breast Cancer Chemotherapy Response
Source: PLoS One. 2013 Dec 9;8(12):e81784. doi: 10.1371/journal.pone.0081784 (PMC3857210; doi:10.1371/journal.pone.0081784)
Supplement: Text S1 — (PDF) [file pone.0081784.s012.pdf]

Supplement

Supplemental Text

Detailed discussion of the ExprEssence subnetwork

In this section, the interactions defining the subnetwork obtained by ExprEssence will be discussed, except for the ones already featured in the main text. The pioneer transcription factor FOXA1 opens the chromatin and allows estrogen and anti-estrogens to bind the DNA [1]. FOXA1 expression is a signal for proliferation of ER-positive breast cancer and an indicator for good outcome of hormone therapy, as are ESR1 and GATA3 [2]. However, with our data, response to neoadjuvant TFAC treatment was examined. Since patients did not receive anti-estrogens, the proliferative effect of FOXA1 that mediates estrogen binding [3] means that downregulation of the interaction between FOXA1 and ESR1 indicates good response under TFAC therapy. Lower FOXA1 gene expression has also been correlated with lower TFF1 protein levels and prevention of hormone-induced reentry into the cell cycle [4]. Further, the lower activity of the interaction between TFF1 and TFF3 in responders matches the hypothesis that high levels of the Trefoil factors promote both tumor growth and migration [5, 6]. Therefore, the downregulation of the interaction path FOXA1–TFF1–TFF3 indicates good response under TFAC therapy.
Among the interactions that are downregulated in responders to TFAC therapy but are not part of the subnetwork highlighted by the green line in Figure 1, we found GFRA1–ISR1. This interaction is part of a RET oncogene related pathway, which mediates IRS1 activation through GFRA-signalling proteins [7]. The lower activity of this growth-related oncogenetic pathway and lower IRS1 levels in responders may slow down breast cancer progression. IRS1 has also been described to be involved in breast cancer tumorigenesis [8].
In concordance with high expression levels of AGR2 being associated with decreased survival [9], we also observe lower amounts of AGR2 in responders. The interaction between AGR2 and LYPD3 was considered a "viable target for oestrogen-responsive breast cancer intervention" by Fletcher et al. [10]. Moreover, according to Maslon et al. [11], RUVBL2 is "overproduced in a panel of primary breast cancer biopsy specimens" and a validated interactor of AGR2. Thus, we suggest that RUVBL2, AGR2 and LYPD3 are forming a module whose downregulation is beneficial and indicative of good prognosis in case of TFAC therapy. The interaction between PLAT and CPB1 is downregulated in responders, but we have no indication of its role based on the literature.
Next, we will focus on the interactions that are upregulated in responders but are not part of the red box in Figure 1. Among them, we observed the interactions KRT7–KRT16 and KRT15–KRT17 to be upregulated, where only the former is differentially regulated in a statistically significant way. We found no consistent biological interpretation for this observation.
The upregulation of SERPINH1 (also known as HSP47) may contribute to a good treatment response by its stimulating effect on procollagen secretion [12, 13], which evokes a protective barrier around cancerous cells. Before cancer cells can migrate to distant sites, this obstacle has to be overcome and therefore the ability to metastasize is decreased in case of collagen secreting cells [14]. Some other serpins are also differentially regulated, but their relevance for breast cancer therapy is unclear.
In addition, we observe an upregulation of S100A1 and S100A7 in responders. Both genes belong to the S100 gene family, which is implicated in breast cancer and melanoma, metastasis, Alzheimer's disease, cardiomyopathy and other diseases. S100A7 was found to be expressed in high-grade ductal carcinoma in situ (DCIS), a key stage to invasive breast cancer [15]. S100A7 is associated with poor prognostic markers in DCIS and influences progression of breast carcinoma through its interaction with the c-Jun activation domain-binding protein 1 (Jab1) [16], thereby enhancing survival under conditions of cellular stress, such as anoikis [17]. Its downregulation was also shown to inhibit EGF-induced cell migration and invasion in

ER-negative MDA-MB-468 cells [18, 19]. Some recent studies, however, also identified tumor-suppressive effects of S100A7 in ER-positive breast cancer cells [20, 21]. High expression of S100A1, as observed among the group of responders, leads to significant reduction of motility and invasion rates in cells expressing also high levels of metastasis-promoting S100A4 [22]. This observation suggests an antagonistic role of S100A1 against S100A4-mediated metastasis [23]. However, it remains unclear whether S100A1 also counteracts tumorigenic effects mediated by S100A7.

References

1. Taube JH, Allton K, Duncan SA, Shen L, Barton MC (2010) Foxa1 functions as a pioneer transcription factor at transposable elements to activate afp during differentiation of embryonic stem cells. J Biol Chem 285: 16135–16144.

2. Jacquemier J, Charafe-Jauffret E, Monville F, Esterni B, Extra JM, et al. (2009) Association of gata3, p53, ki67 status and vascular peritumoral invasion are strongly prognostic in luminal breast cancer. Breast Cancer Res 11: R23.

3. Eeckhoute J, Carroll JS, Geistlinger TR, Torres-Arzayus MI, Brown M (2006) A cell-type-specific transcriptional network required for estrogen regulation of cyclin d1 and cell cycle progression in breast cancer. Genes Dev 20: 2513–2526.

4. Laganire J, Deblois G, Lefebvre C, Bataille AR, Robert F, et al. (2005) From the cover: Location analysis of estrogen receptor alpha target promoters reveals that foxa1 defines a domain of the estrogen response. Proc Natl Acad Sci U S A 102: 11651–11656.

5. Westley BR, May FEB (2006) Identification of steroid hormone-regulated genes in breast cancer. Methods Mol Med 120: 363–388.

6. Dignass A, Lynch-Devaney K, Kindon H, Thim L, Podolsky DK (1994) Trefoil peptides promote epithelial migration through a transforming growth factor beta-independent pathway. J Clin Invest 94: 376–383.

7. Morandi A, Plaza-Menacho I, Isacke CM (2011) Ret in breast cancer: functional and therapeutic implications. Trends Mol Med 17: 149–157.

8. Shaw LM (2011) The insulin receptor substrate (irs) proteins: at the intersection of metabolism and cancer. Cell Cycle 10: 1750–1756.

9. Brychtova V, Vojtesek B, Hrstka R (2011) Anterior gradient 2: a novel player in tumor cell biology. Cancer Lett 304: 1–7.

10. Fletcher GC, Patel S, Tyson K, Adam PJ, Schenker M, et al. (2003) hag-2 and hag-3, human homologues of genes involved in differentiation, are associated with oestrogen receptor-positive breast tumours and interact with metastasis gene c4.4a and dystroglycan. Br J Cancer 88: 579–585.

11. Maslon MM, Hrstka R, Vojtesek B, Hupp TR (2010) A divergent substrate-binding loop within the pro-oncogenic protein anterior gradient-2 forms a docking site for reptin. J Mol Biol 404: 418–438.

12. Hendershot LM, Bulleid NJ (2000) Protein-specific chaperones: the role of hsp47 begins to gel. Curr Biol 10: R912–R915.

13. Ragg H (2007) The role of serpins in the surveillance of the secretory pathway. Cell Mol Life Sci 64: 2763–2770.

14. Fields GB (1991) A model for interstitial collagen catabolism by mammalian collagenases. J Theor Biol 153: 585–602.

15. Enerbck C, Porter DA, Seth P, Sgroi D, Gaudet J, et al. (2002) Psoriasin expression in mammary epithelial cells in vitro and in vivo. Cancer Res 62: 43–47.

16. Emberley ED, Alowami S, Snell L, Murphy LC, Watson PH (2004) S100a7 (psoriasin) expression is associated with aggressive features and alteration of jab1 in ductal carcinoma in situ of the breast. Breast Cancer Res 6: R308–R315.

17. Emberley ED, Niu Y, Curtis L, Troup S, Mandal SK, et al. (2005) The s100a7-c-jun activation domain binding protein 1 pathway enhances prosurvival pathways in breast cancer. Cancer Res 65: 5696–5702.

18. Paruchuri V, Prasad A, McHugh K, Bhat HK, Polyak K, et al. (2008) S100a7-downregulation inhibits epidermal growth factor-induced signaling in breast cancer cells and blocks osteoclast formation. PLoS One 3: e1741.

19. Armstrong DK, Kaufmann SH, Ottaviano YL, Furuya Y, Buckley JA, et al. (1994) Epidermal growth factor-mediated apoptosis of mda-mb-468 human breast cancer cells. Cancer Res 54: 5280–5283.

20. Deol YS, Nasser MW, Yu L, Zou X, Ganju RK (2011) Tumor-suppressive effects of psoriasin (s100a7) are mediated through the -catenin/t cell factor 4 protein pathway in estrogen receptor-positive breast cancer cells. J Biol Chem 286: 44845–44854.

21. Yu SE, Jang YK (2012) The histone demethylase lsd1 is required for estrogen-dependent s100a7 gene expression in human breast cancer cells. Biochem Biophys Res Commun 427: 336–342.

22. Zhang S, Wang G, Liu D, Bao Z, Fernig DG, et al. (2005) The c-terminal region of s100a4 is important for its metastasis-inducing properties. Oncogene 24: 4401–4411.

23. Wang G, Zhang S, Fernig DG, Martin-Fernandez M, Rudland PS, et al. (2005) Mutually antagonistic actions of s100a4 and s100a1 on normal and metastatic phenotypes. Oncogene 24: 1445–1454.
